# Dim Red Light During Scotophase Enhances Mating of a Moth Through Increased Male Antennal Sensitivity Against the Female Sex Pheromone

**DOI:** 10.3389/fgene.2021.611476

**Published:** 2021-02-24

**Authors:** Qiuying Chen, Xi Yang, Dongrui You, Jiaojiao Luo, Xiaojing Hu, Zhifeng Xu, Wei Xiao

**Affiliations:** ^1^Key Laboratory of Entomology and Pest Control Engineering, College of Plant Protection, Southwest University, Chongqing, China; ^2^Academy of Agricultural Sciences, Southwest University, Chongqing, China; ^3^State Cultivation Base of Crop Stress Biology for Southern Mountainous Land of Southwest University, Southwest University, Chongqing, China

**Keywords:** dim red light, scotophase, mating enhancement, *Conogethes punctiferalis*, electroantennography, odorant binding proteins

## Abstract

Insects are behaviorally and physiologically affected by different light conditions, including photoperiod, light intensity, and spectrum. Light at night has important influences on nocturnal insects, including most moth species. Moth copulation and mating usually occur at night. Although a few studies examine changes in insect mating under artificial light at night, detailed influences of light, such as that of monochromatic light, on moth mating remain largely unknown. In this study, on the basis of long-term insects rearing experience, dim red light (spectrum range: 610–710nm, with a peak at 660nm; 2.0 Lux) during scotophase was hypothesized to enhance mating in the yellow peach moth, *Conogethes punctiferalis*. To test the hypothesis, the mating of moths under dim red, blue, and white lights during scotophase was observed. Under the dim red light, the enhancement of mating in *C. punctiferalis* was observed. In addition, the electroantennografic response of males against the female sex pheromone increased with red light treatment during scotophase. In an analysis of the differentially expressed genes in the antennae of males under red light and dark conditions, the expression levels of two odorant-binding protein (OBP) genes, *CpunOBP2* and *CpunPBP5*, were up-regulated. Two genes were then expressed in *Escherichia coli*, and the recombinant proteins showed strong binding to female pheromone components in fluorescence-binding assays. Thus, the results of this study indicated that dim red light at night enhanced the mating of *C. punctiferalis*. One of the mechanisms for the enhancement was probably an increase in the antennal sensitivity of males to the female sex pheromone under red light that was caused by increases in the expression levels of pheromone-binding protein genes in male antennae.

## Introduction

Light influences many behaviors of insects, including host-finding, aggregation, mating, and oviposition (Matthews and Matthews, 2010). Different light conditions have different influences on insect mating behaviors. First, a prolonged photophase usually inhibits mating. For example, in *Heteroplcha jinyinhuaphaga*, when the photoperiod is changed from 6Light∶18Dark to 22Light∶2Dark, calling and mating behaviors, including total calling percentage, onset time of calling, total mating percentage, and mating duration, decreased significantly (Xiang et al., 2018). In *Cnophalocrocis medinalis*, calling frequency decreased substantially under constant light, compared with a normal photoperiod of 15Light∶9Dark (Kawazu et al., 2011). Second, the wavelength of light also influences insect mating behaviors. For example, males of the cabbage butterfly *Pieris rapae crucivora* were more active in UV-rich environments, and they searched longer for females and approached them preferentially in the shade and then copulate there more frequently (Obara et al., 2008). In the tephritid fruit fly *Anastrepha ludens*, males exposed to red, blue, or shaded light treatments in the photoperiod were more frequently chosen as mating partners than dark-reared males, whereas females reared in blue light and darkness mated less compared with those reared in red and shaded light (Diaz-Fleischer and Arredondo, 2011). In a comprehensive study on the influences of light on the reproductive performance of the potential natural enemy *Propylea japonica*, the light intensity, wavelength and photoperiod had important effects on the mating behaviors of pursuit time, number of copulations, and duration of copula (Wang et al., 2014).

In addition to those studies that focus on the effects of photoperiod, intensity, and wavelength on insect mating behaviors, the effect of light in scotophase on insect mating behavior has also recently been investigated. Lifetime exposure to a high level of white light (100 lux) at night increased the probability of a successful mating in the cricket *Teleogryllus commodus* (Botha et al., 2017). In the oriental tobacco budworm *Helicoverpa assulta*, a dim white light (0.5 lux) during scotophase promoted mating, whereas a high-intensity light (50.0 lux) suppressed calling behavior, pheromone production, and mating (Li et al., 2015). Compared with many laboratory experiments, few field tests have been conducted on the influence of light on insect mating at night. van Geffen et al. (2015) investigated the effects of artificial lights (white, green, and red, with intensities <10 lux) at night on the mating of a geometrid moth, *Operophtera brumata*, in an oak-dominated forest in Wageningen, the Netherlands. In their study, fewer mated females were caught on artificially illuminated trees than on the dark control. In addition, fewer males were attracted to a synthetic sex pheromone trap on illuminated trees than to one on the control (van Geffen et al., 2015). These authors concluded that artificial light at night inhibits mating in the moth, although the details of mating under artificial light conditions were not directly observed.

Light in scotophase can also change gene expression levels in insects. For example, with 3h of low light treatment at the beginning of scotophase, the expression of 16 genes decreased and that of 14 genes increased in male adults of the mosquito *Culex pipiens* (Honnen et al., 2016). In the heads of *Helicoverpa armigera* males, the genes *IMFamide*, *leucokinin*, and *sNPF* were differentially expressed between UV-A light (365nm) treatments and the control (Wang et al., 2018).

The yellow peach moth *Conogethes punctiferalis* (Guenée; Lepidoptera: Crambidae) is widely distributed in Asia and Australia. The larvae damage many economically important orchard crops, spices, and vegetables (Xu et al., 2014). Female sex pheromone of the specie includes (*E*)-10-hexadecenal, (*Z*)-10-hexadecenal, (*Z*3, *Z*6, *Z*9)-tricosatriene and (*Z*9)-heptacosene (Konno et al., 1982; Xiao et al., 2011, 2012). In male antennae, chemosensory genes, including *CpunPBP2*, *CpunPBP5*, *CpunGOBP1*, and *CpunGOBP2*, have been demonstrated of pheromone binding function in recent years (Ge et al., 2018; Jing et al., 2019). Since 2012, a colony of the moth has been reared in our laboratory. On the basis of our long-term rearing experience and personal communications (Dr. Ballal, Indian Council of Agricultural Research), the continuous provision of a dim red light during scotophase was hypothesized to enhance mating in the yellow peach moth. Therefore, in this study, the mating behavior of *C. punctiferalis* was firstly observed under red light during scotophase. When the enhancement of mating under red light was confirmed, electroantennography (EAG) tests were conducted to test the antennal responses of male moths reared under red light or in the dark to female sex pheromone components. Finally, the expression levels of chemosensory genes in the antennae of male moths under those two light conditions were compared and the functions of the differentially expressed genes were investigated.

## Materials and Methods

### Insects

A colony of yellow peach moths was started from larvae collected in chestnut orchards in Chongqing, China (N 28°40'38”, E 115°50'20”). In the lab, larvae were reared on chestnuts or corn (Xiao et al., 2012). Pupae were sexed, and the sexes were kept in separate cages at 25 ± 1°C and 40–60% relative humidity under a 15Light∶9Dark photoperiod. A fluorescent lamp provided light in photophase. During scotophase, no light was provided unless operations were necessary, which were illuminated with a 15W red, incandescent lamp. Adults were provided with 10% sugar solution on cotton pads. A fresh apple wrapped in cheesecloth was hung from the top of the cage to collect eggs.

### Chemicals

The synthetic pheromone components including (*E*)-10-hexadecenal (E10-16∶Ald), (*Z*)-10-hexadecenal (Z10-16∶Ald), (*Z*3, *Z*6, *Z*9)-tricosatriene (Z3, Z6, Z9-23∶HC) and (*Z*9)-heptacosene (Z9-27∶HC) were purchased from Shanghai Udchem Technology Co., Ltd., China. N-phenyl-1-naphthylamine (1-NPN) was purchased from Sigma-Aldrich (purity ≥95%; Shanghai, China). All chemicals were stored as specified by the manufacturers.

### Mating and Oviposition Observations

Pairs of newly eclosed adults (1-day-old, male∶female, 1∶1, 30 pairs) were put in a metal cage (50cm × 50cm × 50cm). In each treatment, three cages of insects were treated as replicates. Temperature, photoperiod, and adult food were the same as those for rearing. For use in the light experiments during the 9h of scotophase, red (spectrum range: 610–710nm, with a peak at 660nm), blue (spectrum range: 410–510nm, with a peak at 460nm), and white (spectrum range: mixed) light LED lamps (Shenzhen Ladeng Lighting Technology Co., Ltd., Shenzhen, China) were set approximately 3m above the cages. The intensity of light was approximately 2.0 lux at the middle point of the inner cage space. As a control, no light was provided during scotophase (dark treatment). The number of mating pairs (including both continuously mating and newly mating pairs) was recorded every hour from the 2nd day after eclosion. A video camera (HDR-CX560V in nightshot mode; Sony, Tokyo, Japan) was used to assist with observations in the dark treatment. In an additional red-light experiment, two other light intensities, 0.2 and 20.0 lux, were examined, in addition to 2.0 lux. A spectrometer (HR-350, Highpoint Corporation, Taiwan, China) and an illuminometer (Z-10, Everfine Corporation, Hangzhou, China) were used to measure the light spectra and intensities, respectively.

The cheesecloth with deposited eggs was changed daily, and the number of eggs on the cheesecloth was counted immediately.

### Electroantennography

The EAG technique was applied as described by Beck et al. (2014) under a normal laboratory conditions illuminated with a fluorescent lamp (light intensity >1000 lux). The antennae of 3-day-old virgin males reared under the same conditions as those in the red-light experiment were cut at the base and the distal part of the flagellum tip amputated. Then, an excised antenna was quickly mounted on an electrode fork holder (Syntech, kirchzarten, Germany) with the flagellum tip on the recording electrode and the base end on the reference electrode. The fork holder with excised antenna was immediately connected to a pre-amplifier and placed 1.0cm inside the open end of a glass tube provided with continuous airflow. An air stimulus controller (CS-55, Syntech, kirchzarten, Germany) generated continuous, humidified airflow (400ml/min) or pulsed airflow (280ml/min, 0.5-s pulse duration) for odor delivery. Twenty microliters of each stimulus dissolved in hexane were loaded on a rectangular filter paper (5mm wide and 50mm long), which was subsequently inserted into the wide section (8-mm diameter) of a Pasteur pipette. The pipette containing the stimulus was then inserted with its narrow end into a side port in the wall of the glass tube. Stimuli were tested with the solvent hexane tested as the blank at the start of a stimuli series. Each concentration of a stimulus was tested once with an antenna. The EAG amplitudes from four antennae of different moths were recorded as replicates for a stimulus. The analog signal of the antennal response was detected through a probe (INR-II, Syntech, kirchzarten, Germany), captured and processed with an Intelligent Data Acquisition Controller (IDAC-4, Syntech, kirchzarten, Germany), and analyzed using EAG 2000 software (Syntech, kirchzarten, Germany) on a PC. The dose of each synthetic pheromone component was tested as a ratio of a female equivalent (FE). For each of *E*10-16∶Ald, *Z*10-16∶Ald, *Z*3, *Z*6, *Z*9-23∶HC and *Z*9-27∶HC, one FE was 9.55, 0.45, 2.4 and 270ng (Xiao et al., 2011, 2012).

### Expression Levels and Functions of Antennal Chemosensory Genes

#### Reverse-Transcription Quantitative PCR of Chemosensory Genes

Based on the transcriptome sequencing (unpublished) of the antennae of 3-day-old male adults with red light and dark treatments during scotophase, six odorant-binding protein (OBP) genes were identified with expression levels that were significantly up-regulated by the red-light treatment. Then, the expression levels of those genes were verified by reverse-transcription quantitative PCR (RT-qPCR), primers showed in Table 1. Primer efficiency was tested using 3-fold diluted cDNA samples, and a standard curve was generated. The Ct values were plotted against the log of the cDNA dilutions, and the efficiency percentage and *R*^2^ values were within the acceptable range. A two-step program was adopted. The reaction volume was set to 20μl, which included 10μl of SYBR Premix ExTaq mixture (Takara Biotechnology Co., Ltd., Dalian, China), 1μl of each of the forward and reverse primers (concentration: 10μM), 1μl of cDNA, and 7μl of RNase free water. The program was designed as follows: denaturation at 95°C for 2min, followed by 39cycles at 95°C for 15s and 60°C for 30s, with melting curve analysis performed from 60°C to 95°C to determine the specificity of PCR products. Three independent biological replicates were processed for all samples. The 2^−∆∆CT^ method was used to calculate the relative expression of genes according to that of the reference gene RP49 (GenBank number: KX668533; Yang et al., 2017).

**Table 1:** The forward and reverse primers of quantitative PCR (qPCR).

Gene	Forward primer	Reverse primer
GOBP1	TACTTCAACCTGATCACCGA	CCTCCTCGAACTGCTTCT
PBP5	GTCAACGTACGAGTGGAAGC	CTCCCCTAGCTCAGAGTCCT
OBP2	ATGTTGGCGTGTTGGATGAC	CCGAGTTGACTGGAGAGCAA
OBP3	AGGAGTTAGAGTCGATTGCA	ATTTCAGAGCCAATATCGCC
OBP11	TCTTTTAGTGGTGTGGTGTGT	CTGGATTCACGTTCGTCTCC
OBP13	GTGCATGGATGACGAGATG	GCACTTGATGTAGCACTTGA

#### Preparation of Recombinant Proteins

The signal peptides of *CpunOBP2* and *CpunPBP5* (GenBank number: KF026055.1 and KP985227, respectively) were predicted by the SignalP server (Petersen et al., 2011). Then, primers were designed with signal peptides removed from the complete open reading frame sequences. With antennal cDNA used as the templates, *CpunOBP2* and *CpunPBP5* were amplified and cloned using the Pclone-007s vector (TsingKe Biotech, Beijing, China). The following forward and reverse primers were designed for *CpunOBP2* and *CpunPBP5* according to homologous recombination as Table 2 shown.

**Table 2:** The forward and reverse primers were designed according to homologous recombination of *CpunOBP2* and *CpunPBP5*.

*CpunOBP2*	forward primer	GGCCATGGCTGATATCGGATCCATGACAGAGGAACAAAGA
	reverse primer	CTCGAGTGCGGCCGCAAGCTTTTAGA CGTCGATGCCAA
*CpunPBP5*	forward primer	GGCCATGGCTGATATCGGATCCTCTCAGGA GGTGATGAAGAA
	reverse primer	CTCGAGTGCGGCCGCAAGCTTCTAGGCTTCACCTATCATCT

The expected gene sequences were purified and cloned into the bacterial expression vector pET-32a (+), (TsingKe Biotech, Beijing, China) digested with the same enzymes. Then, the plasmid was transformed into BL21 (DE3) competent cells (TsingKe Biotech, Beijing, China), and colonies were grown on LB ampicillin agar plates. A single positive clone was first identified and then grown in liquid LB with ampicillin overnight at 37°C. The culture was diluted to 1∶100 and cultured for 5–6h at 37°C until its absorbance at OD (600nm) reached 0.6–0.8. To induce the protein, isopropyl-β-D-thiogalactoside (Solarbio, Beijing, China) was added to the culture at a final concentration of 0.1mM, and the culture was incubated at 15°C and 150r.min^−1^ for 18h. The induced bacterial cells were harvested from a volume of 200ml of liquid LB medium and centrifuged at 4°C for 20min (4,000 *g*). The pellets were subjected to ultrasonication and were centrifuged at 4°C for 20min (9,000 *g*) to obtain the soluble proteins, and the recombinant protein were purified by the Ni^2+^-IDA column (His tagged) with a gradient concentration imidazole washing. The western blot method was performed to analyze the correct expression. Ten microliters of purified protein sample were electrophoresed in 10% gel (BIO-RAD, Shanghai, China). The gel was sandwiched with a fluoride polyvinylidene fluoride (PVDF) membrane, and the sample in the gel was transferred to the PVDF (electrophoresis; 240mA, 1h). The film was washed three times using Tris-HCl buffer solution-Tween (TBST; Cebio, Beijing, China). Then, 5% skim milk powder in TBST was used as the blocking buffer for 1h and then washed three times by TBST. The PVDF membrane was incubated with the primary antibody His (1∶1,000) overnight at 4°C. The membrane was washed three times again using TBST and treated with the secondary antibody sheep anti-rabbit antibody (1∶10,000) at room temperature for 1h. After a final wash, the membrane was developed in Gel imager (Analytik Jena, Jena, Germany) using Super ECL reagent (ELC kit, Coolaber, Beijing, China). The purified protein was obtained with tags removed by enterokinase from recombinant proteins.

#### Fluorescence-Binding Assay

A fluorescence-binding assay was used to measure the affinity of the recombined CpunOBP2 and CpunPBP5 to female sex pheromone components (Liu et al., 2012; Ge et al., 2018). An F970CRT fluorescence spectrophotometer (Lengguang Technology Co., Ltd., Shanghai, China) with a 1-cm light path fluorimeter quartz cuvette was used for the assay. The fluorescent probe 1-NPN and all tested chemicals were dissolved in methanol of HPLC-grade purity, and the final concentration of chemicals used in the assay was 1mM. To measure the affinity of the fluorescent ligand 1-NPN to each protein, a 2μM solution of the protein in 30mM Tris-HCl, pH 7.4, was titrated with aliquots of 1mM ligand in methanol to final concentrations of 2–20μM. The fluorescence of 1-NPN was excited at 337nm, and emission spectra were recorded between 350 and 500nm. The affinity of chemicals was measured in competitive binding assays, using 1-NPN as the fluorescent reporter at a concentration of 2μM and each chemical at different concentrations from 0.5 to 8μM (Liu et al., 2013; Sun et al., 2013; Hua et al., 2020).

GraphPad Prism 8 (GraphPad Software, Inc., San Diego, CA, United States) was used to estimate the K_1-NPN_ (K_d_ of complex protein / 1-NPN) values by nonlinear regression for a unique site of binding. The proteins were assumed to be 100% active, with a stoichiometry of 1∶1 (protein∶ ligand) at saturation. For other competitor ligands, the dissociation constants were calculated from the corresponding IC_50_ values (concentration of a ligand halving the initial fluorescence value of 1-NPN) using the following equation: K_i_ = [IC_50_]/(1 + [1-NPN]/K_1-NPN_). In the equation, [1-NPN] is the free concentration of 1-NPN, and K_1-NPN_ is the dissociation constant of the complex protein/1-NPN (Ge et al., 2018; Jing et al., 2019).

### Statistical Analyses

Data from different light (red, blue, and white) experiments were analyzed by independent sample *t*-tests. Data from different intensities of red light were subjected to the least significant difference test after ANOVA. Absolute EAG amplitudes of the blank were subtracted from the EAG amplitude of the stimuli (Ngumbi et al., 2010). Then, the data were analyzed by ANOVA, and the least significant difference test was used for multiple comparisons of means. Oviposition and gene expression data were analyzed by independent sample *t*-tests.

## Results

### Mating Pairs Under Different Conditions During Scotophase

**Figure 1:**
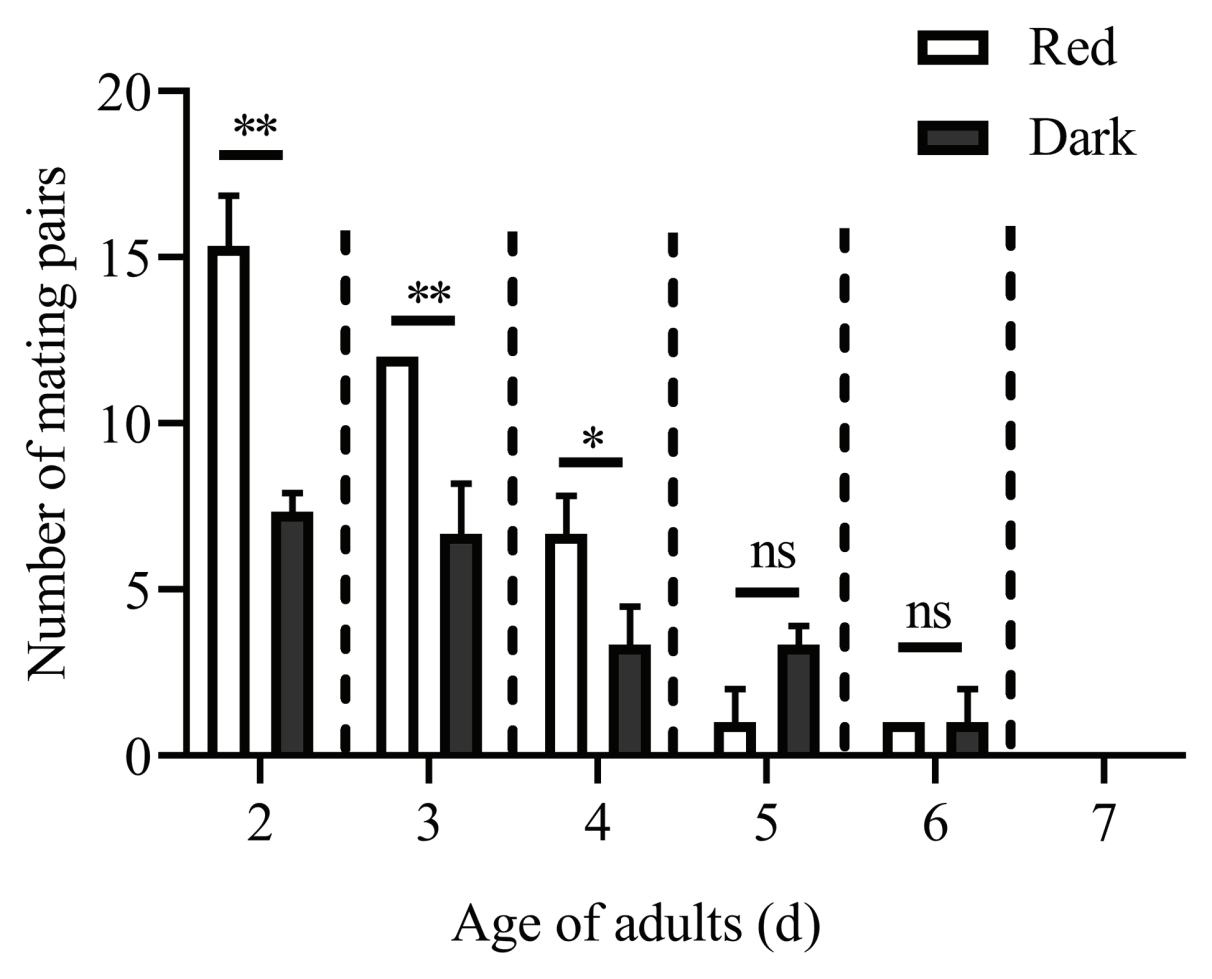
Mating pairs of *Conogethes punctiferalis* under red light and dark conditions during scotophase. Data are shown as mean ± SD, from three replicates. Asterisks above columns mean significant differences (^*^p < 0.05, ^**^p < 0.01, ns: no significant difference), by independent sample *t*-tests.

The numbers of mating pairs of *C. punctiferalis* under red light and dark conditions in scotophase during 6 days after adult eclosion are shown in Figure 1. Most of the mating occurred in the 2 to 4-day-old adults under both light conditions. The number of mating pairs under red light was significantly higher than that in the dark on each of first 3days. In the five and 6-day-old adults, the number of mating pairs was not significantly different under the two light conditions. In the 7-day-old adults, no mating was observed under either light condition. The oviposition of females is shown in Table 3. The numbers of eggs laid by 3 to 5-day-old females under red light were significantly higher than those in the dark. Meanwhile, the egg numbers under red light on those 3days accounted for approximately 76% of the total recorded numbers during 5days. In addition, the average number of eggs laid per female per day under red light was significantly higher than that in the dark.

**Table 3:** Oviposition of *C. punctiferalis* under red light and dark conditions during scotophase.

Lights	Age of adults (d)					Eggs /♀/d
	2	3	4	5	6	
Red light	311.0 ± 174.5a	1055.0 ± 121.8a	1046.0 ± 13.8a	951.0 ± 85.9a	626.0 ± 169.8a	26.6 ± 2.13a
Dark	277.0 ± 231.8a	520.0 ± 57.0b	635.0 ± 103.5b	681.0 ± 83.1b	677.0 ± 276.5a	18.3 ± 3.5b

Data are shown as mean ± SD, from three replicates. Data with different letters in the same column are significant different, *p* < 0.05, by independent sample *t*-tests.

**Figure 2:**
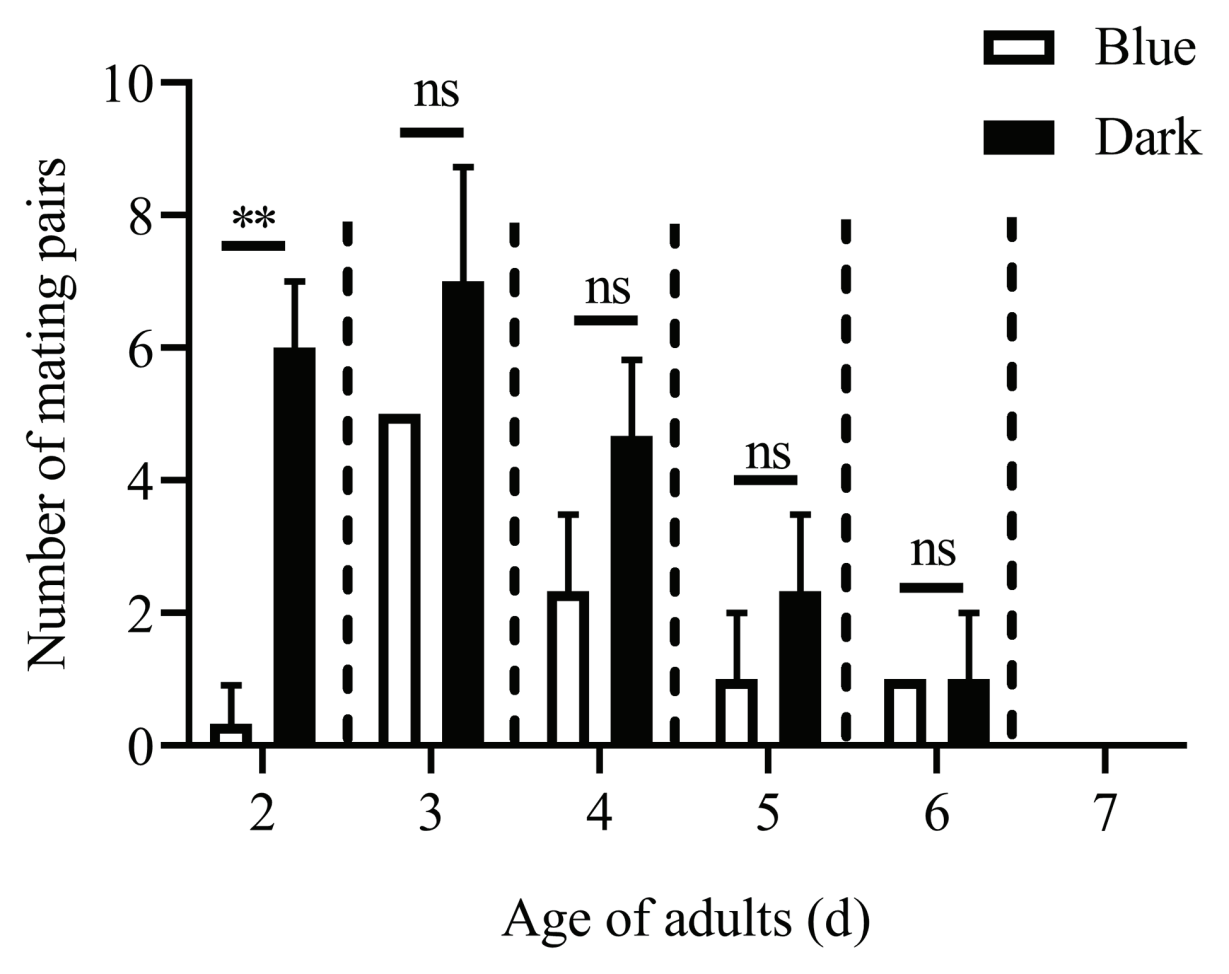
Mating pairs of *C. punctiferalis* under blue light and dark conditions during scotophase. Data are shown as mean ± SD, from three replicates. Asterisks above columns mean significant differences (^**^p < 0.01, ns: no significant difference), by independent sample *t*-tests.

**Figure 3:**
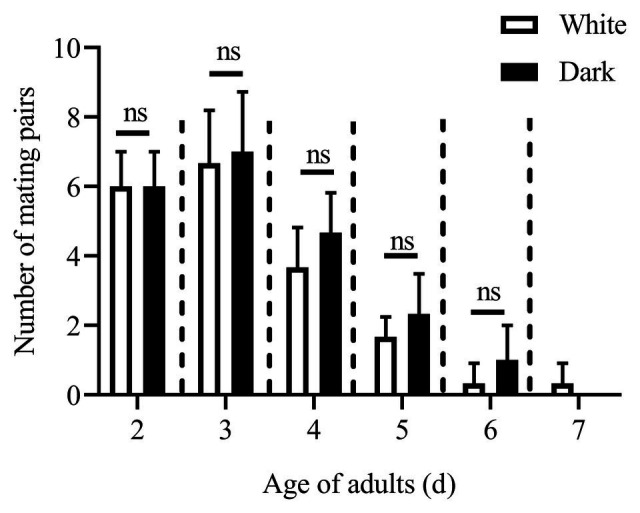
Mating pairs of *C. punctiferalis* under white light and dark conditions during scotophase. Data are shown as mean ± SD, from three replicates. Asterisks above columns means significant differences (*p* < 0.05, ns: no significant difference), by independent sample *t*-tests.

Except for the 1st day, the numbers of mating pairs of *C. punctiferalis* were not significantly different between blue light and dark conditions in scotophase during 6days of observation (Figure 2). On the 1st day, the number of mating pairs in blue light was significantly lower than that in the dark (Figure 2). A similar result was found in the white light experiment. There were no significant differences in numbers of mating pairs between white light and dark conditions during 6days of observation (Figure 3).

### Effects of Different Intensities of the Red Light on Mating

**Figure 4:**
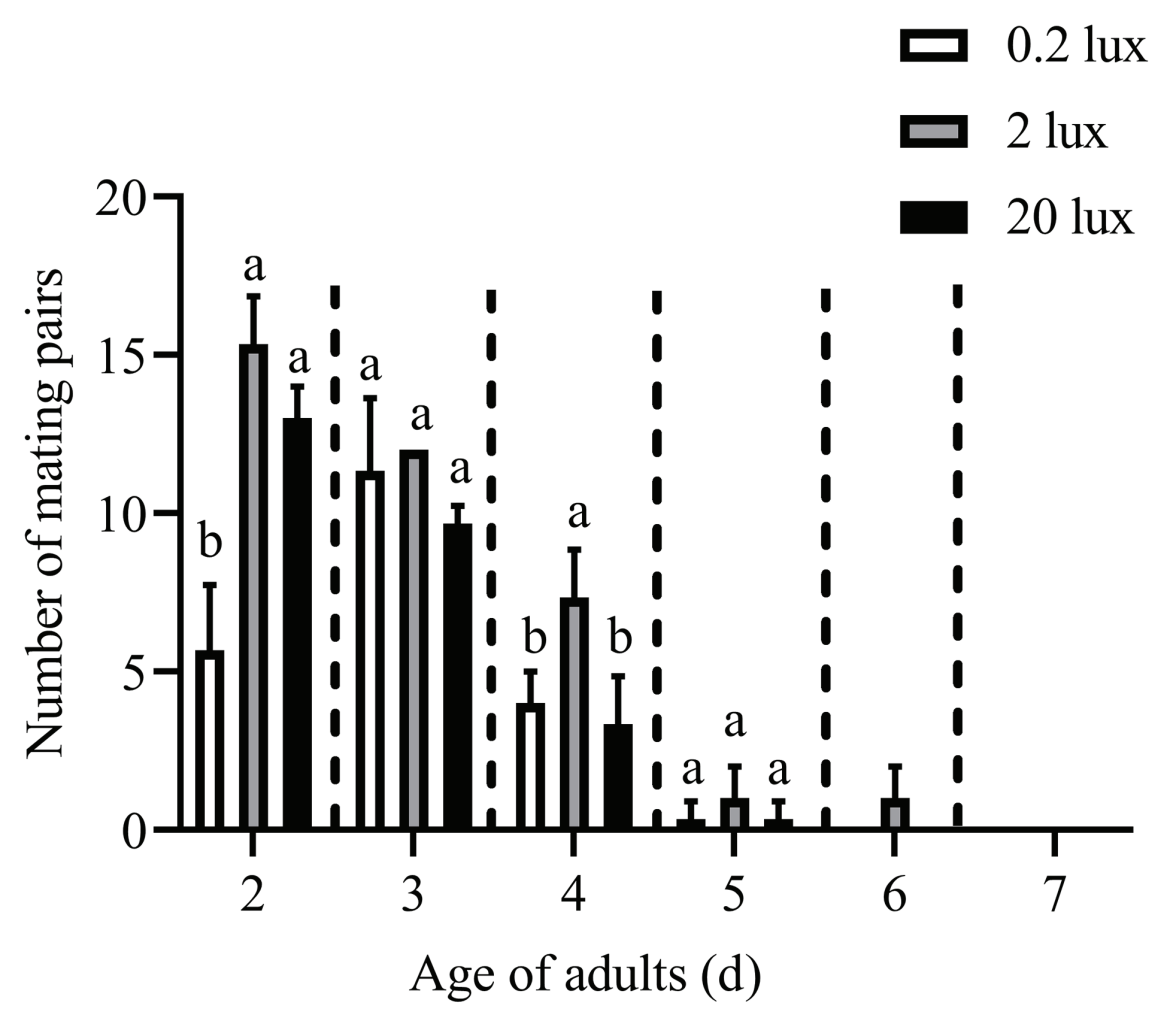
Mating pairs of *C. punctiferalis* under different intensities of red light during scotophase. Data are shown as mean ± standard deviation, from three replicates. Different letters above columns mean significant differences (*p* < 0.05, ns: no significant difference), by Tukey’s multiple comparisons after ANOVA.

The effects of different intensities of red light on the number of mating pairs of *C. punctiferalis* in scotophase are shown in Figure 4. Similar to the results in Figure 1, most of the mating was observed in the first 3days under all light intensities. In the 2-d-old adults, the number of mating pairs under 2.0 and 20.0-lux red light was significantly higher than that under 0.2 lux. No differences in the number of mating pairs were observed among the three light intensities for 3-d-old adults. In the 4-day-old adults, the number of mating pairs under the 2.0-lux red light was significantly higher than that under 0.2 and 20.0 lux. In addition, no differences were observed in the number of mating pairs among the three intensities in the 5-day-old adults. No mating was observed in the 6 to 7-day-old adults except a few mating was found under 2.0-lux red light in the 6-day-old adults.

### EAG Responses

**Figure 5:**
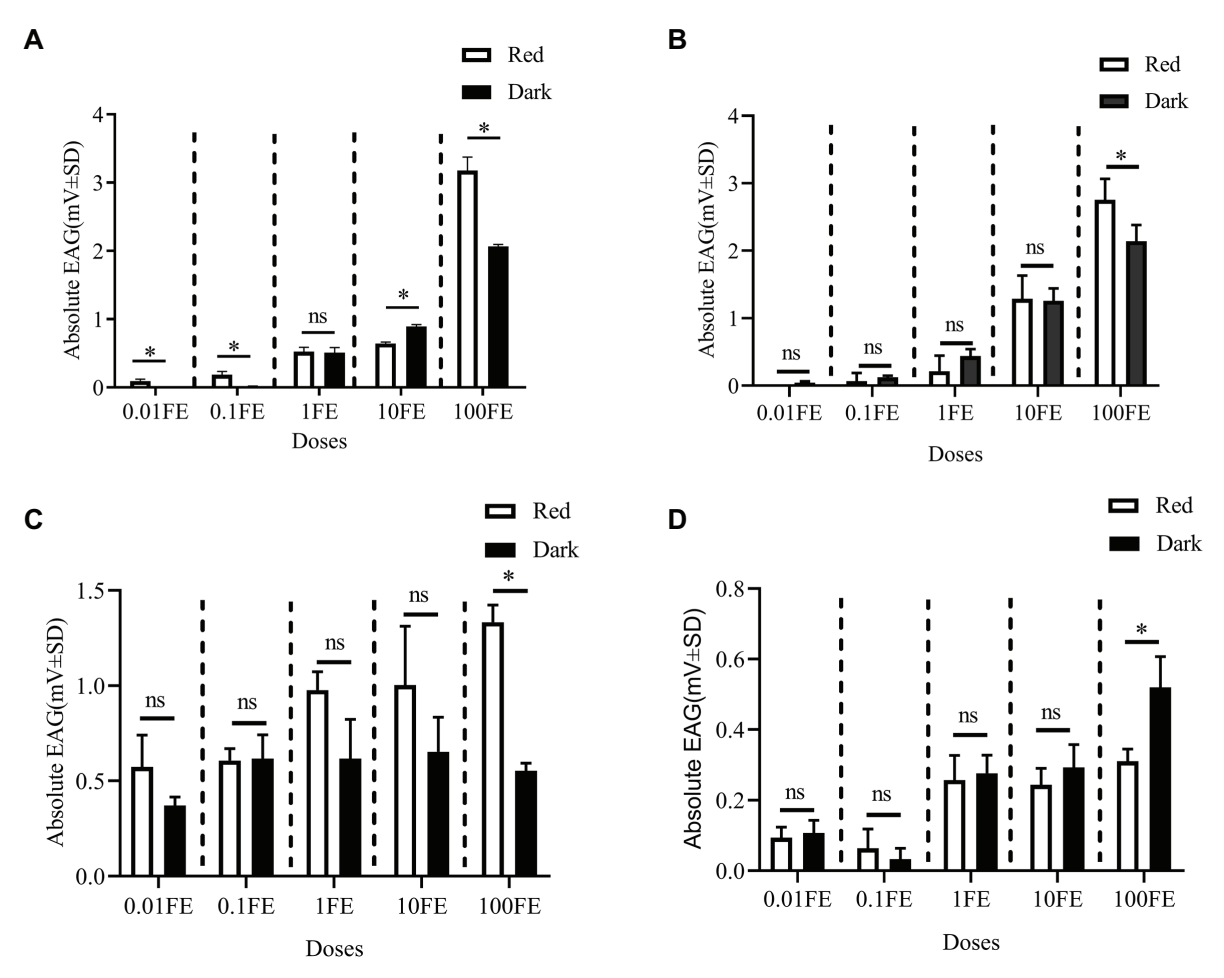
Electroantennographic (EAG) responses of male *C. punctiferalis* reared under red light and dark conditions during scotophase. **(A)** E10-16∶Ald; **(B)** Z10-16∶Ald; **(C)** Z9-27∶HC Z6; and **(D)** Z3, Z6, Z9-23∶HC. Data are shown as mean ± SD, from four replicates. Asterisks above columns mean significant differences (^*^p < 0.05, ns: no significant difference), by independent sample *t*-tests. FE, female equivalent.

**Figure 6:**
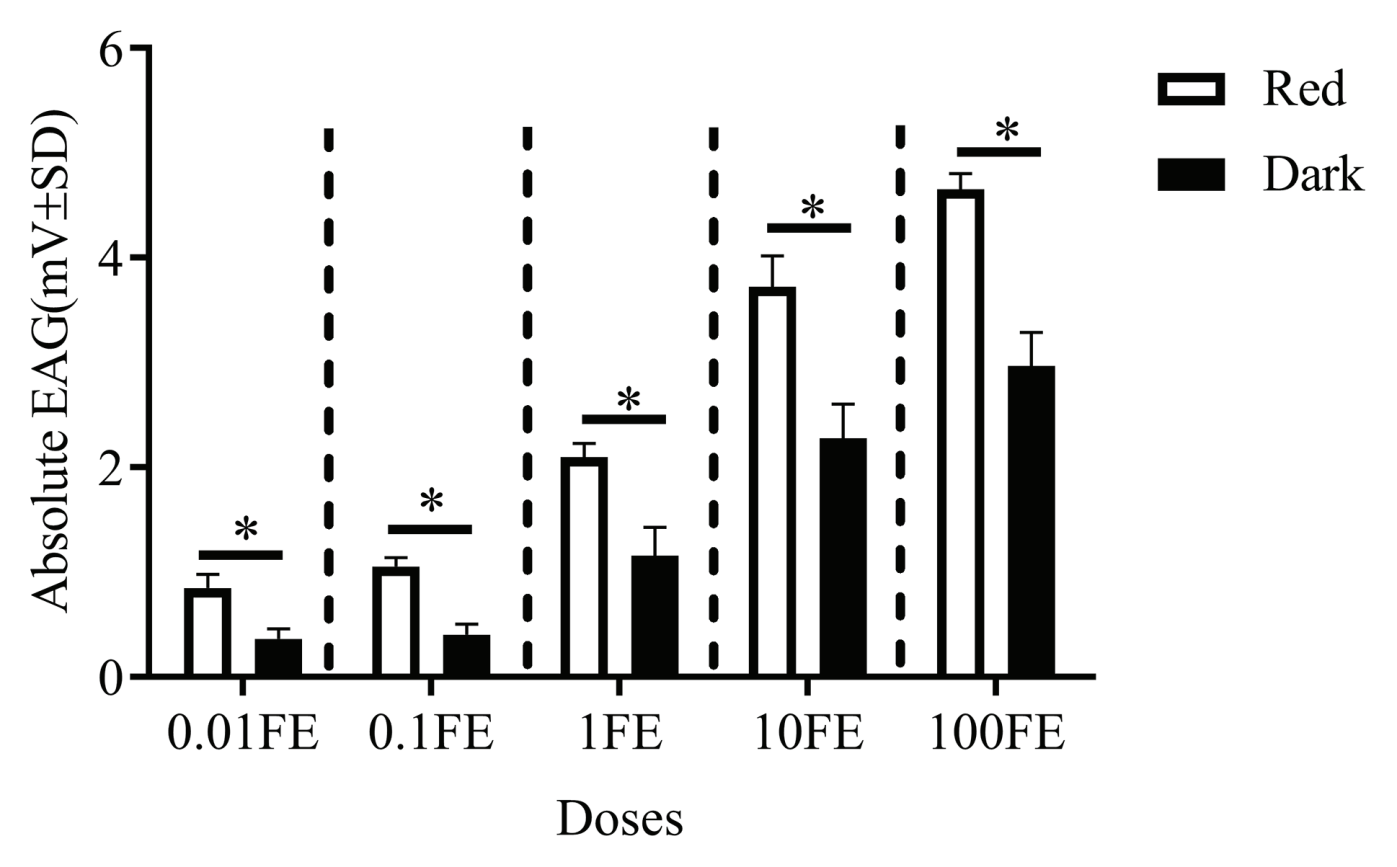
Electroantennographic (EAG) responses to the mixture of sex pheromones of male *C. punctiferalis* reared under red light and dark conditions during scotophase. Data are shown as mean ± SD, from four replicates. Asterisks above columns mean significant differences (^*^p < 0.05, ns: no significant difference), by independent sample *t*-tests. FE: female equivalent.

Figure 5 shows the EAG responses of male adults reared under red light and dark conditions during scotophase to the single female pheromones component (Figures 5A–D) and the pheromone mixture (Figure 6). For E10-16∶Ald (Figure 5A), among the five tested doses of E10-16∶Ald (Figure 5A), the EAG responses of male moths under red light were significantly higher than those of males in the dark at doses of 0.01, 0.1, and 100 FEs, respectively. However, at the dose of 10 FE, the EAG response of males in the dark was higher than that of males under red light. By contrast, for all doses of Z10-16: Ald except of 100FE (Figure 5B), there were no differences in EAG responses at other tested doses between male antennae under red light and in the dark. Additoanlly, for Z9-27∶HC, the EAG responses of male moths under red light were much higher than that in the dark at the only one dose, 100FE, among five tested doses (Figure 5C). While, for Z3, Z6, Z9-23∶HC, no difference of the EAG responses between male moths under red light and in the dark was found at the tested dose, except of 100FE, at which the EAG response of male moths in the dark was significantly higher than that under red light (Figure 5D). Finally, when all pheromone components were mixed and tested, the EAG response of male moths under red light was significantly higher than that in the dark at each of all tested doses (Figure 6).

### Expression Levels of Genes

**Figure 7:**
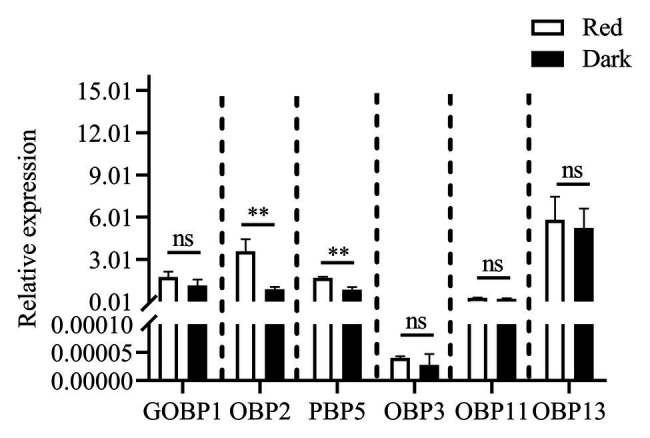
Expression levels of odorant binding protein genes, in antennae of *C. punctiferalis* males (3-day-old) under red light and dark conditions during scotophase. Data are shown as mean ± SD, from three replicates. Asterisks above columns mean significant differences (^**^p < 0.01, ns: no significant difference), by independent sample *t*-tests.

Reverse-transcription quantitative PCR was performed to determine the expression levels of six differentially expressed genes (under red light and dark conditions during scotophase) screened from the transcriptome analysis as shown in Figure 7, the relative expression of *CpunOBP2* and *CpunPBP5* in antennae of males under red light was significantly higher than that in the dark.

### Fluorescent Ligand-Binding Assays

**Figure 8:**
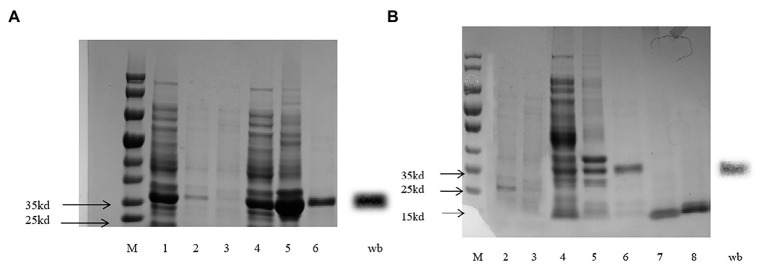
SDS-PAGE for recombined CpunPBP5 **(A)** and CpunOBP2 **(B)**.

**Figure 9:**
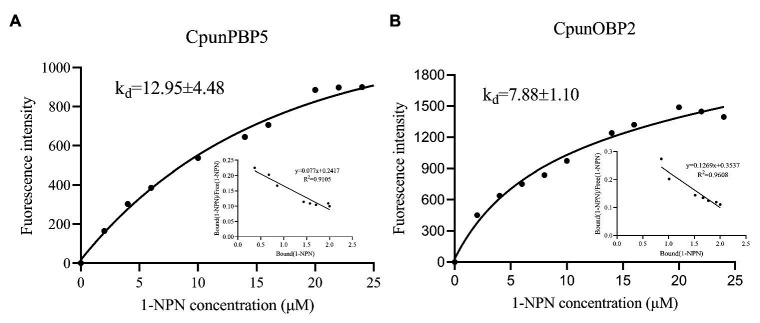
Binding curve and relative Scatchard plot for 1-NPN/CpunOBP2 **(A)** and 1-NPN/CpunPBP5 **(B)**.

**Figure 10:**
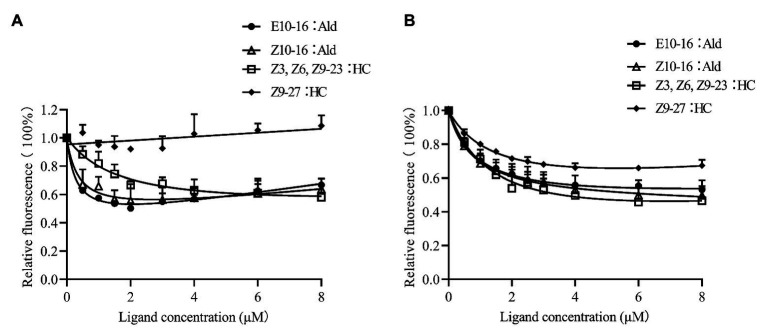
Competitive binding curves of CpunPBP5 **(A)** and CpunOBP2 **(B)** to sex pheromones. Data are shown as mean ± SD, from three replicates.

*CpunOBP2* and *CpunPBP5* were expressed in *Escherichia coli*, and the recombined protein (about 1mg/ml) was purified by affinity chromatography. The presence of recombinant CpunOBP2 and CpunPBP5 was checked by SDS-PAGE (Figure 8). With tags removed by enterokinase, proteins were then subjected to a fluorescence displacement-binding assay. Based on the binding curves and the Scatchard plots (Figure 9), the dissociation constant of the proteins/1-NPN complex was calculated as 7.88 ± 1.10μM for *CpunOBP2* and 12.95 ± 4.48μM for *CpunPBP5*. Then, the binding affinity of the proteins to the female sex pheromones E10-16∶Ald, Z10-16∶Ald, Z9-27∶HC and Z3, Z6, Z9-23∶HC was measured. As shown in Figure 10, the intensities of recombinant CpunOBP2 decreased with the increase in ligand concentration (E10-16∶Ald, Z10-16∶Ald and Z3, Z6, Z9-23∶HC). And for recombinant CpunPBP5, the lowest decrease of intensity was found in the ligand of E10-16∶Ald, among four pheromone components. Then, the IC_50_ and K_i_ values of the two proteins were calculated (Table 4). The binding affinity of the recombined CpunOBP2 was 8.48 ± 4.06μM to E10-16∶Ald, 6.86 ± 2.50μM to Z10-16∶Ald and 3.68 ± 2.33μM to Z3, Z6, Z9-23∶HC (Table 4), which indicated the protein bound to the three pheromone components with a similar and relatively high affinity. Meanwhile, CpunOBP2 showed no binding to Z9-27∶HC. By contrast, the binding affinity of CpunPBP5 was 1.47 ± 0.62μM to E10-16∶Ald, which indicated much stronger binding affinity of the protein to the ligand, compared with that of CpunOBP2. However, to Z10-16∶Ald Z3, Z6, Z9-23∶HC, and Z9-27∶HC, the recombined CpunOBP5 showed no binding affinity (Table 4).

**Table 4:** IC_50_ values (μM) and dissociation constants (K_i_; μM) of CpunOBP2 and CpunPBP5 to different sex pheromones at pH = 7.4. Data are shown as mean ± SD, from three replicates.

Proteins	Ligand	IC_50_ (μM)	K_i_ (μM)
CpunOBP2	E10-16∶Ald	9.55 ± 4.58	8.48 ± 4.06
	Z10-16∶Ald	7.73 ± 2.82	6.86 ± 2.50
	Z3, *Z*6, *Z*9-23∶HC	4.14 ± 2.63	3.68 ± 2.33
	Z9-27∶HC	-	-
CpunPBP5	E10-16∶Ald	1.58 ± 0.67	1.47 ± 0.62
	Z10-16∶Ald	-	-
	Z3, Z6, Z9-23∶HC	-	-
	Z9-27∶HC	-	-

## Discussion

When artificially rearing moths, dim red light is usually used to aid observations during scotophase. In this study, dim red light at night was hypothesized to increase mating in *C. punctiferalis*. To test this hypothesis, mating of *C. punctiferalis* was observed during scotophase with and without dim red light (610–710nm, 2.0 lux). Many more adults were observed mating in red light compared with those in the dark. However, most mating was not enhanced under dim blue or white light. In addition, under different intensities of red light (0.2, 2.0, and 20.0 lux), similar enhancement in mating was observed. Moreover, the increased antennal sensitivity to female sex pheromones was found in male adults reared under red light during sctotophase. In a subsequent analysis, upregulation of expression levels of two OBP genes was determined in antennae of males under red light. The pheromone-binding affinities of the proteins of those two genes were confirmed in fluorescent-binding assays.

Insects are generally assumed to be essentially blind to red wavelengths (Shimoda and Honda, 2013). Therefore, red lights are frequently used to aid observations or operations while working with artificially reared insect populations during scotophase, because the influence on insects is negligible. However, different behaviors of insects were reported with or without red light. For example, red wavelengths influenced aggregation in the ant *Lasius niger*, and among colony individuals, foragers aggregated well in total darkness but showed low assembly under red light, whereas brood-tenders aggregated well in both conditions (Depickere et al., 2004). The sexual performance of a lekking tephritid fruit fly (*A. luddens*) is also affected by red light (Diaz-Fleischer and Arredondo, 2011). Male flies exposed to red light were more frequently chosen as mating partners than dark-reared males. Similarly, females reared in red light mated more than those reared in blue light and in darkness.

Note that in the above publications, red lights were provided as a light source only in photophase, and no light was provided during scotophase. The effects of weak light on insect behavior in scotophase are rarely studied. Especially for many moth species, mating of which usually occurs at night. In the oriental tobacco budworm *H. assulta*, mating was increased by a dim white light (0.5 lux) during scotophase (Li et al., 2015). However, in that study, a 15W incandescent bulb provided the light, which was probably broad-spectrum light rather than monochromatic one. In addition, the moths were held in cages with one pair (male and female) per cage. As a result, the increase in mating was observed at only one time point, i.e., 1h into scotophase. In this study, a monochromatic red light with the spectrum of 610–710nm was used as the light source. Meanwhile, there were 30 pairs of males and females per cage (with three replicates), which gave individuals relatively free mate choice during mating. Thus, the increase in mating under red light during several consecutive scotophases was clearly observed in this study (Figure 1).

The EAG responses of male *C. punctiferalis* antennae under red light to single pheromone component, except of Z3, Z6, Z9-23∶HC, was much higher than that of males in darkness, at the highest tested dose of 100FE (Figures 5A–D). When four pheromone components were further mixed and tested, the EAG response of male moths under red light was found increased significantly, compared with that in the dark at each of all tested doses (Figure 6). Generally, the sex pheromone of a Lepidopterous specie is composed of multi-components (Ando, 2018). Antennal responses of males usually could be elicited by a single component and the mixture of components. In natural environment, a male individual perceives female sex pheromone components as a mixture in spite of single ones. Therefore, the pheromone mixture elicits more comprehensive antennal response of males, compared with the single component does. In our results, obvious difference in EAG response between males under red light and in darkness against female sex pheromone was found when all pheromone components were tested as a mixture rather than singe ones. These results clearly indicate that red light enhances the antennal sensitivity of males to the female sex pheromone. The olfactory perception of female sex pheromones plays an essential role in the pheromone communication system of moths (Stengl, 2010). A variety of molecular components, including OBPs, chemosensory proteins, and odorant receptors, are involved in peripheral sensory reception and signal transduction in insects (Suh et al., 2014). Therefore, when the expression levels of chemosensory genes are up-regulated, increases in EAG responses are reasonably detected (Wan et al., 2015). In this study, the increase in the pheromone sensitivity of male adults with red light treatment was most likely due to the up-regulation of two OBP genes, i.e., *CpunOBP2* and *CpunPBP5*. The recombined proteins of these two genes, especially for CpunOBP2, showed strong binding to female sex pheromone components (Figures 10A,B). Of the two OBPs, CpunPBP5 has been previously identified as one of the pheromone-binding proteins in *C. punctiferalis* (Ge et al., 2018), whereas pheromone-binding affinity of CpunOBP2 was reported for the first time in our study.

Pre-exposure to various pheromones and plant volatiles frequently changes the expression levels of chemosensory genes in insect antennae (Wan et al., 2015). At the transcriptional level, artificial light at night was recently found to influence gene expression in *H. armigera* and *C. pipiens* (Honnen et al., 2016; Wang et al., 2018). In this study, the expression of OBP genes in male antennae of *C. punctiferalis* was up-regulated by red light during scotophase, with verification by RT-qPCR. With this finding, this study is the first to report that the expression of chemosensory genes in insects can be changed by monochromatic light. However, mechanisms of gene expression changed by red light remain largely unknown. Red light can penetrate animal tissues because of long wavelength (Fitzgerald et al., 2013; Zer-Krispil et al., 2018). And the cytochrome c oxidase of the mitochondrial respiratory chain is considered as the photoacceptor for the red light (Karu, 2008; Lunova et al., 2019). Irradiation of mammalian cells with red light causes an upregulation of various genes, most of which directly or indirectly play roles in the enhancement of cell proliferation and the suppression of apoptosis (Zhang et al., 2003). In our study, whether up-regulated expression of the two OBP genes in *C. punctiferalis* male antennae is relative to cytochrome *c* oxidase activated by red light is yet to be investigated.

In summary, we found that a dim red light enhanced mating of the yellow peach moth, *C. punctiferalis*, while a dim blue light and white light did not. Meanwhile, the red light increased expression of pheromone binding protein genes, *CpunOBP2* and *CpunPBP5*, in antennae of male adults, which showed increased antennal responses to female pheromone components. In brief, the increase in the antennal sensitivity of males to the female sex pheromone is probably one of the mechanisms for the increase in mating in *C. punctiferalis* under the dim red light during scotophase.

## Data Availability Statement

The raw data supporting the conclusions of this article will be made available by the authors, without undue reservation.

## Author Contributions

ZX and WX designed the research and wrote the article. QC, XY, DY, JL, and XH performed the experiments. All authors contributed to the article and approved the submitted version.

### Conflict of Interest

The authors declare that the research was conducted in the absence of any commercial or financial relationships that could be construed as a potential conflict of interest.
